# A 15-Year One Health Approach to Antimicrobial Resistance in Kuwait from Hospitals to Environmental Contexts: A Systematic Review

**DOI:** 10.3390/life15091344

**Published:** 2025-08-25

**Authors:** Ahmad Al-Dhumair, Mohammad Al-Hasan, Hanan Al-Khalaifah, Qadriya Al-Mutawa

**Affiliations:** Food Security Program, Kuwait Institute for Scientific Research, Safat, Kuwait City 13109, Kuwait; mhasan@kisr.edu.kw (M.A.-H.); hkhalifa@kisr.edu.kw (H.A.-K.); qmutawa@kisr.edu.kw (Q.A.-M.)

**Keywords:** antimicrobial resistance, Kuwait, humans, animals, environment, One Health

## Abstract

Kuwait has reported a problematic increase in the prevalence of Antimicrobial Resistance (AMR). However, the absence of studies that analyze AMR from combined human, agricultural (animal), and environmental domains limits our ability to assess the extent of the problem in Kuwait. Therefore, this systematic review provides a comprehensive insight into the AMR status in Kuwait regarding humans, agriculture (animals), and the environment from the perspective of the One Health approach. A systematic search was conducted according to the Preferred Reporting Items for Systematic Reviews and Meta-Analyses (PRISMA) guidelines to identify the relevant literature on AMR in Kuwait. Multiple online electronic databases, including the Cochrane Library, Google Scholar, Web of Science, PubMed, BioMed Central (BMC), and Scopus, were searched to perform a narrative synthesis and meta-analysis. Twenty-eight studies published between 2009 and 2024 were included in this study. Domain-wise distribution varied, with 11 studies related to clinical settings, 11 to the environment, 4 to agricultural (animal), and 2 to both clinical and community settings. The narrative synthesis indicated a high occurrence of AMR bacteria in human, agriculture (animal), and environmental domains. In human domains, the dominant AMR isolates belonged to four Gram-negative species: *E. coli*, *K. pneumoniae*, *P. aeruginosa*, and *Acinetobacter baumannii*. In agriculture (animals), *Salmonella* isolates from poultry display high resistance to cefotaxime, ampicillin, and amoxicillin. Camel milk analyses revealed that 80% of bacterial isolates are resistant to antibiotics such as penicillin, tetracyclines, and carbapenems. An environmental analysis of sewage, seawater, sediment, and aerosol samples documented widespread antibiotic resistance genes (ARGs) with resistance mechanisms such as extended-spectrum beta-lactamase, carbapenemases, and colistin. The cross-domain analysis identified the overlapping of ARGs. Regarding the One Health approach, none of the studies used this approach to interlink these sectors. Meanwhile, the meta-analysis indicated a high resistance rate in humans (34.05%, 95% CI (22.81 to 46.27, *p* < 0.0001, I^2^ = 98.94%)), agriculture (animals) (67.42%, 95% CI (30.30 to 94.93, *p* < 0.0001, I^2^ = 97.40%)), and environment (69.86%, 95% CI (48.80 to 87.26, *p* < 0.0001, I^2^ = 98.78%)). The reported spread of AMR and the overlap of resistance genes among isolates across the domains demonstrate the interconnected nature of AMR in Kuwait. These findings underscore the need to adopt the One Health approach to strengthen surveillance, implement control measures, and enhance public education strategies to address the complexity of AMR challenges in Kuwait.

## 1. Introduction

Antimicrobial agents are successfully used in the healthcare sector to control infections and have saved millions of human lives [1]. Likewise, these agents are used in agriculture (animals) to control infections, prevent disease, and promote growth [2]. However, their irrational use facilitates the development of antimicrobial resistance (AMR), which has become a health concern on a global scale [3]. The development of AMR emerges when microbes, including bacteria, fungi, and parasites, evolve to resist the effects of commonly used antimicrobial agents [4]. AMR imposes a serious burden on healthcare systems worldwide in terms of morbidity, mortality and healthcare expenditures [5,6,7]. In 2019, the impact of AMR bacteria on morbidity and quality of life was significant, as they were associated with 2.29 million Years Lived with Disability (YLDs) [5]. Similarly, 4.95 million deaths were associated with infections caused by AMR bacteria [5]. The death toll is expected to increase globally to reach 59.7/100,000 in 2021 to 87.7/100,000 in 2050 [6,8]. AMR also has a negative impact on global economy. The analyses estimate that the annual global cost of AMR, including healthcare expenditures and productivity loss, is between USD 100 and 150 billion and is projected to increase to USD 300 billion by 2030 [7].

The development of AMR is an evolutionary adaptive process that slowly occurs in nature under normal environmental conditions in response to the selective pressure of antimicrobials [9,10,11]. However, the misuse of antimicrobials is the main driving force for the rapid emergence of AMR, as it increases the selective pressures and thus dramatically accelerates the process [11]. These selective pressures develop from the inappropriate, excessive, or unnecessary use of antimicrobials in human healthcare and veterinary practices, as well as from the environmental release of chemical stressors. In the human sector, the selective pressure developed from inappropriate prescribing, self-medication, and the incomplete treatment of infectious diseases. Similarly, selective pressures from the animal sector are produced by the use of mass medication practices to treat infections, prevent diseases, and promote growth. Additionally, the environmental release of antimicrobials, heavy metals, and disinfectants increases selective pressure on the microbial community. Collectively, the increase in selective pressures from human activities across domains accelerates the development of AMR [11]. Moreover, there is increasing concern over the role of the environment, particularly water bodies, as they act as reservoirs for AMR and play a critical role in the dissemination of antimicrobial resistance genes (ARGs) [12,13]. Although there is limited evidence that the environment has been directly affected by AMR, an unhealthy environment, such as water pollution or the spread of microbes via fecal waste, can increase the risk of AMR in humans and animals [14]. In addition, environmental drivers have a significant influence on the emergence and spread of AMR. Those drivers include ambient conditions [15,16,17,18,19], heavy metals [20], and microplastics [21]. Multiple studies reported that AMR is strongly influenced by environmental conditions such as oxygen availability, acidity and alkalinity, and temperature [15,16,17,18,19]. Oxygen availability influences the abundance of ARGs. Fan et al. [15] showed that aerobic conditions reduce tetracycline resistance genes (TRGs) in activated sludge by 33.2%, whereas anaerobic conditions increase the abundance of those genes. The impact of pH on ARGs varies and depends on the environment and the type of ARGs [16]. Acidic pH reduces some ARGs, such as sulfonamide-resistant genes (sul) in manure, while increasing others, such as TRGs in sludge. On the contrary, alkalinity often reduces the abundance of ARG [16]. The fluctuations in temperature due to seasonal and climate changes strongly influences AMR development. The rise in water temperature from 5 °C to 30 °C increases the frequency of the conjugate transfer of plasmid-mediated ARGs 30-fold [17]. Heavy metal contamination is an important co-selector for AMR. A study conducted in the soil and the rhizosphere of plants grown in mercury-contaminated sites highlighted that mercury contamination indirectly contributes to the spread of AMR [20]. Mercury promotes the co-selection of ARGs that confer resistance to different antibiotics in *Bacillus* spp. In total, 72% of the isolates were resistant to multiple antibiotics, including ampicillin, benzylpenicillin, cephalosporins (cefotaxime and ceftriaxone), and tetracyclines [20]. Microplastics act as vectors for the dissemination of ARG. Peng et al. [21] identified 18 different ARGs on 21 microplastic polymers detected in indoor dust samples. These ARGs confer resistance to macrolides, sulfonamides, tetracyclines, β-lactams, aminoglycosides, and amphenicols. The hydrophilicity and bioavailability properties of the polymers determine their influence on ARG enrichment. Polymers with hydrophilic and bioavailable properties provide ideal surfaces for bacterial growth and biofilm formation and thus facilitate the spread of ARGs [21].

To combat AMR globally, several international organizations, such as the Food and Agriculture Organization (FAO), the World Organization for Animal Health (WOAH), and the World Health Organization (WHO), have formed a partnership to develop and implement a Global Action Plan (GAP) [22]. The main goal of the GAP is to preserve, to the greatest extent possible, the ability to treat and prevent infectious diseases using safe and effective medicines through responsible use, while ensuring their continued accessibility to those in need [22]. It emphasizes the importance of a comprehensive One Health approach by including human and veterinary medicine, agriculture, finance, environmental agencies, and informed consumers [22]. Therefore, the GAP provides integrated guidelines for all Member States to follow and implement to address AMR using a coordinated approach [22].

The Middle East and North Africa (MENA) region struggles with serious AMR challenges. The reported resistance levels across the region are higher than the global average. In 2019, 256,000 deaths were associated with AMR, while 63,300 deaths were directly linked to bacterial resistance [5]. The reported resistance to certain pathogen–drug combinations in the region was among the highest globally. For example, the resistance prevalence of methicillin-resistant *Staphylococcus aureus* (MRSA) in countries such as Iraq and Kuwait was between 60% and 80%, which is higher than the prevalence reported in many other parts of the world [5]. Like other MENA countries, the Gulf Cooperation Council (GCC) countries reported a worrying prevalence of AMR bacteria. In a systematic review of clinical samples from GCC countries, *E. coli* (44%), *K. pneumoniae* (20%), and *P. aeruginosa* (18.7%) were the most frequently isolated species [23]. The resistance prevalence among the isolates varied from one country to another. Kuwait reported the highest resistance prevalence in *E. coli* (77%) and *K. pneumoniae* (36.2%) compared to other GCC countries. On the other hand, Saudi Arabia reported the highest resistance prevalence for *P. aeruginosa* (92.3%) and *Acinetobacter* (83.3%). Bahrain and Oman had the highest resistance prevalence in *Enterococcus* (76.5%) and MRSA (58.3%), respectively. Unlike other countries, the resistance prevalence in clinical isolates from Qatar and the UAE were the lowest among the GCC countries [23]. Worrying AMR levels were also reported in the marine environment; 32.5% of *E. coli* isolates from marine environments of four GCC countries (Kuwait, Oman, United Arab Emirates and Bahrain) were multidrug-resistant (MDR) bacteria. Most of the resistance was reported against ciprofloxacin (44.6%), ampicillin (29.6%), and nalidixic acid (27.9%) [24]. High resistance was reported among the clinical *K. pneumoniae* isolates. Most of the isolates were resistant to cefazolin (64.75%), trimethoprim/sulfamethoxazole (62.63%) and ampicillin (59.45%). Importantly, 48.95% of isolates were extended-spectrum beta-lactamase (ESBL) producers [25]. Additionally, MRSA has been identified in samples obtained from food-producing animals, pets, and food products. The prevalence has been identified in different animal species, including goats (≤28.9%), camels (≤35.5%), and cats (≤44.4%). In food products, MRSA prevalence was reported in camel meat (20%), processed foods (62.6%) and dairy products (72.9%). Some strains share clonal lineages with human-associated MRSA, which suggests intersectoral transmission between humans, animals, and the food chain [26]. In addition, a high prevalence of ESBL and carbapenem-resistant (CR) infections was reported in the GCC countries [27]. In Saudi Arabia, the prevalence of ESBL and CRE ranged from 8.94% to 85% and 35.5% to 88%, respectively. blaCTX-M-15 and blaOXA-48 were the dominant ESBL and CR detected genes. In the UAE, 75% of the community-acquired urinary tract infections were associated with ESBL-producing *E. coli* and *K. pneumoniae*. Most of the isolates were resistant to ciprofloxacin (74%) and trimethoprim/sulphamethoxazole (73%). In Qatar, ESBL and CR isolates are dominant among pediatric populations. blaCTX-M-15 was detected in 87.8% of the ESBL-producing *E. coli* and *K. pneumoniae* isolates. Among CRE isolates, *E. coli* (65.3%) and *K. pneumoniae* (30.6%) were the most common species. The dominant CRE genes detected among isolates were blaNDM-5 (30.8%), blaNDM-1 (19.2%), and blaOXA-181 (19.2%) [27]. The high prevalence of ESBL and carbapenemase genes has been recently documented among *K. pneumoniae* isolates. While blaCTX-M-15 was the most commonly detected ESBL gene across the GCC countries, blaOXA and blaNDM were the dominant identified carbapenemase genes in all countries [28]. According to Rahman et al. [29], 28% of *E. coli* isolates from Arabian oryx in Qatar are resistant to at least one antibiotic, and 2% of those were MDR. Most isolates were resistant to tetracycline (26%) and ampicillin (8%) [29]. Abdalla et al. [30] revealed that all *Vibrio* species isolated from commercially sold fish and shellfish were resistant to penicillin G, daptomycin, and vancomycin. Resistance to ampicillin was lower in shellfish isolates (6–10%) compared to fish isolates. The resistance against sulfamethoxazole-trimethoprim was low in both shellfish (2–12%) and fish (33–40%) isolates [30]. Wang et al. [31] detected new ARG sewage systems after mass religious gatherings (Hajj and Umrah) in Saudi Arabia. Although the abundance of ARGs remains constant, blaPER was introduced into the sewage system and detected in *Shewanella putrefaciens*, *S. xiamenensis*, and *Aeromonas media*. Waste water produced from mass gathering-affected treatment plants exhibited higher variability in resistance profiles compared to those produced from control sites [31].

Kuwait has experienced a worrying increase in the prevalence of AMR. Between 1999 and 2003, Kuwait had an average antibiotic resistance level of 27% for the eight most common pathogens. This level was higher than the resistance average reported by the United States of America (17%) and comparable to that reported by China (28%) [32]. Kuwait reported higher resistance rates than both countries for certain pathogen–antibiotic combinations including *P. aeruginosa* against fluoroquinolones (31%) and ceftazidime (27%), *K. pneumoniae* against ciprofloxacin (18%) and ceftazidime (14%), and *Salmonella* species against ciprofloxacin (10%) [32]. In Kuwait, the distinct socio-economic variables complicate AMR, necessitating a country-specific One Health approach that addressed both clinical and structural drivers [33]. Therefore, the country developed its NAP for combatting AMR in 2022 according to the GAP’s guidelines by involving various sectors, including human and animal medicine, environment, food chain, finance, and the general public [33]. It focused on governance, surveillance, and awareness regarding AMR, the prevention and control of infections, and, most importantly, the rational use of antimicrobials [33]. However, the implementation of the One Health approach in Kuwait is problematic. This is due to the weakness in intersectoral coordination, the gap in multisectoral research studies, the absence of a multisectoral AMR surveillance system, and the insufficiency of evidence-based management across the human, animal, agriculture, and environmental sectors [33]. Due to the limited available pooled data on the multisectoral combined analysis of AMR in Kuwait, it was considered appropriate to present a narrative synthesis of the prevalence data. Thus, this systematic review aimed to provide a comprehensive AMR analysis of the complex interplay between humans, animals, and environmental health sectors over a 15-year period (2009–2024). This study examines the relationship between One Health and AMR in Kuwait. Our thorough study will provide stakeholders and policymakers with a solid basis for informed decision-making in combating AMR in Kuwait. This study addresses the following research questions:

1. What has been the status of AMR in Kuwait over the past 15 years (2009–2024)?
2. How do several sectors, including human health, animal health, and the environment, influence the emergence and dissemination of AMR?
3. What information is available about the efficacy of One Health measures in reducing AMR in Kuwait?

## 2. Materials and Methods

### 2.1. Study Design

This study followed a comprehensive search approach following the Preferred Reporting Items for Systematic Reviews and Meta-Analyses (PRISMA) 2020 guidelines [34] to identify studies examining AMR in Kuwait from humans, animals and the environment. PRISMA Extension for Scoping Reviews was reported (Online Supplementary, Table S1).

### 2.2. Search Strategy

The electronic databases, such as the Cochrane Library, Google Scholar, Web of Science, PubMed, BioMed Central (BMC), and Scopus, were searched to identify relevant AMR studies performed in Kuwait between January 2009 and January 2024. Additionally, relevant grey literature sources such as government reports and conference proceedings were included to ensure the inclusion of all relevant studies. Different key words, such as “antimicrobial resistance” OR “antibiotic resistance” OR “multidrug resistance” OR “resistant pathogens” OR “AMR” AND “one health” OR “interdisciplinary” OR “intersectoral” AND “human health” OR “clinical isolates” AND “animal health” OR “zoonotic” AND “agriculture” OR “farms” AND “environment” OR “water contamination” OR “food chain” OR “soil contamination” were used. Meanwhile, Boolean operators (AND, OR) were used to connect the key words.

### 2.3. Eligibility Criteria

For the selection of studies, the following inclusion and exclusion criteria were used (Table 1).

### 2.4. Study Selection Process

**Figure 1:**
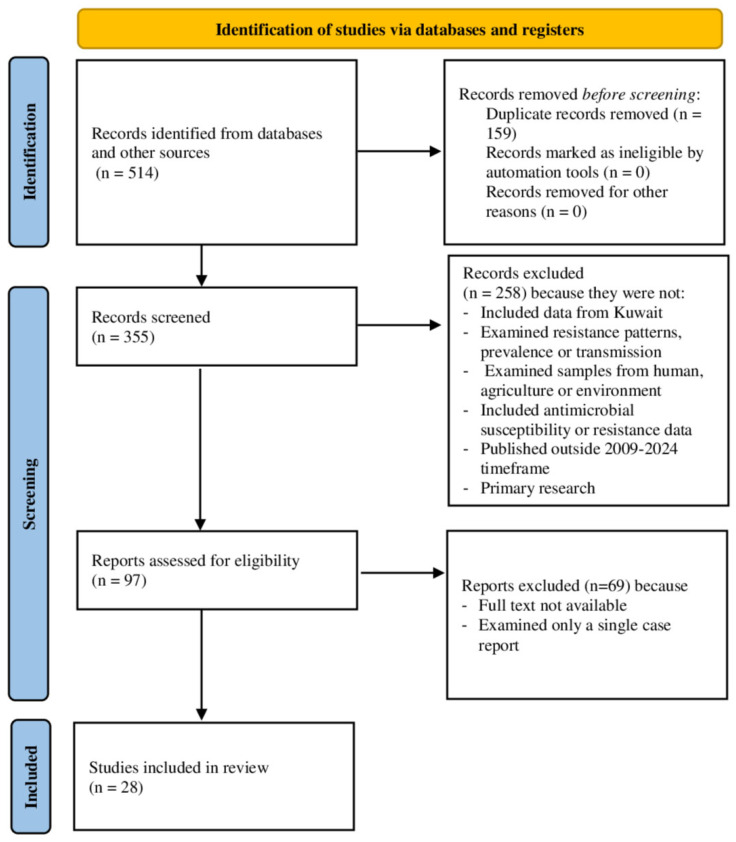
PRISMA flowchart for the selection of studies.

Two independent reviewers used the PRISMA flowchart for the selection of studies. Initially, studies were identified from different electronic databases, and duplicate studies were removed. The remaining studies were screened and assessed according to the titles and abstracts, and irrelevant studies were excluded, while relevant studies were further selected for full-text assessment. Full-text assessment was performed following inclusion/exclusion criteria, and studies that fulfilled the eligibility criteria were included in the systematic review, while the remaining studies were excluded with reasons explained in the PRISMA flowchart (Figure 1). Studies that fulfilled the criteria were qualitatively and quantitatively analyzed. Meanwhile, any discrepancy among the two reviewers was resolved by consulting a senior reviewer.

### 2.5. Data Extraction

Two independent reviewers used a predefined data extraction Microsoft^®^ Excel^®^ sheet (Version 2508 Build 16.0.19127.20082). The reviewers extracted specific details about the study’s geographical and environmental context (specific locations within Kuwait where samples were collected, the types of environments sampled (marine, hospital, agricultural, community), and seasonal variations). Sample characteristics (total number of samples, types of samples (*E. coli* isolates, seawater, biota), source of samples (marine environment, clinical settings), and breakdown of sample types (351 from seawater, 247 from Venus clam)) were recorded. Antibiotic resistance screening (number of antibiotics tested, specific antibiotics included in the screening, resistance rates for each antibiotic, and method of resistance determination) was also documented. Key resistance findings (prevalence of resistance, most common resistant antibiotics, variations in resistance (by season, location, sample type), and potential implications for human, animal, or environmental health) were summarized.

### 2.6. Methodological Quality Assessment

The methodological quality assessment was performed by two independent reviewers. The risk of bias in the non-randomized controlled trials (non-RCTs) was assessed using the Risk of Bias In Non-randomized Studies—Interventions (ROBINS-I) tool, and it was then categorized as low, high, and some concerns [35]. The web-based open access tool Robvis was used for visualization and reporting the assessment outcomes [36]. Animal studies were evaluated using SYRCLE and the risk assessment tool, and each study was assessed in the domains of selection, performance, detection, attrition, reporting, and other biases [37]. The environmental studies were evaluated using an assessment tool based on the Cochrane risk of bias-2 tool [38].

### 2.7. Meta-Analysis

Qualitative data were presented in table form and summarized the key characteristics of studies and patients. Quantitative data were analyzed using a random effect model for the construction of forest plots using MedCalc^®^ Version 23.3.5 (MedCalc Software Ltd., Ostend, Belgium; 2025) [39], and the association was measured using a chi-square test at the significance level of <0.05. Meanwhile, heterogeneity was calculated using I^2^ statistics, and heterogeneity of <25%, 26–75% and >75% was considered to be low, moderate, and high heterogeneity, respectively. Publication bias was calculated using a funnel plot, and the distribution of studies was symmetrical, making a clear funnel shape, which was considered to indicate low publication bias. In the case of the asymmetrical distribution of studies without a funnel shape, higher publication bias is indicated.

### 2.8. Certainty of Evidence

The Grading of Reporting, Assessment, Development, and Evaluation (GRADE) framework was used for the assessment of the certainty of evidence of the outcomes. Two independent reviewers performed the whole process in the domains of risk of bias, inconsistency, precision, indirectness of the outcomes, and publication bias. Each domain was evaluated based on predefined criteria, and the certainty of evidence for each outcome was then rated as low, moderate, or high.

## 3. Results

### 3.1. Literature Searched

Initially, 514 studies were retrieved from different electronic databases, and 159 studies were found to be duplicates and removed. In the screening phase, 355 studies were screened for their titles and abstracts, while 258 studies were excluded due to the reasons described in Figure 1. Of the remaining studies, 97 were selected for full-text assessment, and strict inclusion and exclusion criteria were followed for the final selection of studies. During this process, 69 studies were excluded for various reasons (Figure 1), and 28 studies were included for further qualitative and quantitative analysis.

### 3.2. General Characteristics

The majority of the included studies focused on the human domain (13 studies) including 11 clinical-based studies [40,41,42,43,44,45,46,47,48,49,50], followed by environmental [24,51,52,53,54,55,56,57,58,59,60] and agriculture-associated studies [61,62,63,64]; two studies were performed in mixed settings (clinical/community) [65,66]. The primary focus of the included studies was the prevalence of AMR among the bacterial isolates against different antibiotics, as described in Table 2. Numerous studies also provide insights into the genomic basis of resistant genes and resistomes [50,52,55,58,61]. Most of the studies were performed on environmental sampling and surveillance techniques for reporting the AMR status in Kuwait. Numerous studies followed observational (retrospective), cross-sectional, and prospective study designs, except for three studies, which did not clearly mention their study design [50,60,64]. The sample size varied with a minimum of 2 samples and a maximum of 6922 samples isolated from the included studies [47,60], as indicated in Table 2.

Moreover, Table 3 provides a summary of the key characteristics of the included studies. Overall, 11 studies were performed in clinical settings, 11 were performed in the context of environmental settings, 4 in agricultural (animal) settings, and 2 in a mixed clinical and community setting. All of the studies were performed in Kuwait as a single country, except for one, which was performed across multiple GCC countries (Table 3). The majority of the studies (23) focused on the prevalence of AMR, and 5 studies focused on the resistant genes. Meanwhile, none of the studies explain or incorporate the One Health approach in their research (Table 3).

### 3.3. Resistance Patterns and Distribution

#### 3.3.1. Clinical Resistance Patterns

The most commonly reported AMR prevalences in clinical settings were among the Gram-negative isolates, including *E. coli*, *K. pneumoniae*, *P. aeruginosa*, and *Acinetobacter baumannii*, [40,41,42,43,44,46,66]. The AMR prevalence in *E. coli* isolates was high (>40%) against antibiotics such as ampicillin, cephalosporins, ciprofloxacin, meropenem, and trimethoprim/sulfamethoxazole. Similarly, *P. aeruginosa* exhibited high AMR prevalence (>40%) against piperacillin/tazobactam, ciprofloxacin, meropenem, and trimethoprim/sulfamethoxazole. The MDR prevalence among the isolates was 37.4% and 32.1% for *E. coli* and *P. aeruginosa*, respectively [42].

Another study reported a high incidence of resistance among the Gram-negative bacteria species, including *E. coli*, *K. pneumoniae*, *P. aeruginosa*, and *Acinetobacter baumannii* [43]. In total, 50% of those isolates were resistant to ampicillin, cefotaxime, cefuroxime, ceftazidime, ciprofloxacin, and sulfamethoxazole/trimethoprim. The investigation revealed the highest incidence of MDR in *E. coli* isolates from respiratory specimens (48%), wounds, bones, or other tissues (47.7%), and bodily fluids (47.1%) [43]. The frequency of MDR in *K. pneumoniae*, *P. aeruginosa*, and *A. baumannii* obtained from respiratory specimens was considerably (*p* < 0.05) greater than that in other specimen categories. The investigation determined that the most prevalent MDR phenotypes among the four Gram-negative species and across various specimen types encompassed three classes of antimicrobial agents (penicillins, fluoroquinolones, and cephalosporins) [43]. Furthermore, 26% of *E. coli* and 55% of *K. pneumoniae* were reported to be resistant to cephalothin, ampicillin, nitrofurantoin, clavulanic acid/amoxicillin, and trimethoprim/sulfamethoxazole [40]. Similarly, high resistance (100%) was shown by *K. pneumoniae* against amikacin, cefoxitin, ciprofloxacin, cefoxitin, ertapenem, imipenem, and meropenem. Likewise, *E. coli* isolates also showed resistance (100%) against cefoxitin, ciprofloxacin, ertapenem, imipenem, and meropenem [41]. During the period from 1997–2007, the AMR rates for penicillin increased from 0.6% to 28.7%, while those for ceftriaxone were 0–7% and those for cefotaxime were 0–16.5%; MDR rates increased from 22.4% to 37.8% [46]. In addition, resistance to tigecycline, colistin, imipenem and meropenem was reported in *Acinetobacter* spp. [44]. In total, 13.6% of the *Acinetobacter* isolates were resistant to tigecycline and 12% to colistin. Resistance to imipenem and meropenem was observed in 25.2% and 37.2% of isolates, respectively. Moreover, 88.4% of the isolates were identified as MDR.

High AMR prevalence was reported in a community-based study focused on the fecal isolates of Carbapenem-resistant *Enterobacteriaceae* (CRE), *E. coli* and *K. pneumoniae*. The prevalence of CRE among was 7.7% and most the isolates were resistant to ampicillin (63.3%), tetracycline (41.7%) and cefalotin (40.8%). An alarming prevalence of colistin resistance (11.3%) was also reported. The prevalence of MDR was higher among *E. coli* isolates (30.6%) compared to *K. pneumoniae* (17.5%). Similarly, the prevalence of extended-spectrum β-lactamase (ESBL) production was higher among *E. coli* isolates (18.8%) compared to *K. pneumoniae* isolates (4.0%) [66].

Furthermore, some *S. aureus* isolates were found to be resistant to trimethoprim (33.5%), tetracycline (38.3%), gentamicin (38.5%), fusidic acid (41.2%), clindamycin and erythromycin (42.4%), ciprofloxacin (42.7%), and kanamycin (43%). On the other hand, the isolates were susceptible to linezolid, vancomycin, and teicoplanin [48].

Likewise, healthcare-associated (HA) and community-acquired (CA) *C. difficile* infections (CDIs) were tested against different antibiotics, and the outcomes revealed that all CA isolates exhibited resistance to ampicillin, in contrast to 4.7% of HA isolates. Meanwhile, the HA isolates exhibited greater resistance to clindamycin compared to the CA isolates, with HA isolates demonstrating a resistance rate of 47.4% against 43% for CA isolates (*p* = 0.70). The resistance to erythromycin was more prevalent in HA isolates than in CA isolates. Resistance to imipenem was prevalent in both HA and CA isolates, although resistance to meropenem was more frequent in CA isolates than in HA. Rifampicin and tigecycline resistance were frequently seen in HA isolates relative to CA isolates [65]. Moreover, research also discovered that none of the 168 samples from neonates had MDR *Enterobacteriaceae*. However, 12 (13.6%) of the samples from mothers had resistance. In addition, resistant genes for ESBL, aminoglycosides, fluoroquinolones, and folate pathway inhibitors were also uncovered. However, no resistant genes for beta-lactam–beta-lactamase inhibitor combinations, carbapenems, or tigecycline were found [49]. A summary of resistant patterns among bacterial species in different domains of detection are listed in Table 4.

#### 3.3.2. Agricultural Sources

In total, 80% of *Salmonella enteritidis* isolates from chicken carcass samples were found to be sensitive to amoxicillin/clavulanic acid. On the other hand, 100%, 94%, and 87.8% *S. enteritidis* isolates were resistant to cefotaxime, ampicillin, and amoxicillin alone, respectively. Meanwhile, 60% of the *S. typhimurium* isolates were sensitive to amoxicillin/clavulanic acid. On the other hand, the study showed 100%, 94%, and 96% resistance to cefotaxime, ampicillin, and amoxicillin alone. Similarly, 88% of *S. Thomson* isolates were sensitive to amoxicillin/clavulanic acid. However, these isolates showed 100% resistance to cefotaxime and 98% resistance to both ampicillin and amoxicillin alone. Furthermore, 60% *S. munchen* isolates were sensitive to amoxicillin/clavulanic acid and 100% were resistant to ampicillin, cefotaxime, and culfamethoxazole/trimethoprim. It was also found to be resistant to gentamycin, amoxicillin, spiramycin, and doxycycline, with resistance rates of 96%, 92%, 84%, and 80%, respectively [62].

Sheep meat samples were collected from different locations across Kuwait to assess the presence of antimicrobial residues [63]. Antimicrobial residues tested positive in muscle (82%), liver (64%) and kidney (100%) samples. The quantification analysis indicated that the mean concentrations (µg/kg) of amoxicillin were 45.26, 64.43, and 53.12 in muscle, liver, and kidneys, respectively. For oxytetracycline, the concentrations were 148.17, 263.15, and 368.21 (kidney), while, for tetracycline, they were 103.18 (muscle), 177.04 (liver), and 196.40 (kidney) [63]. Another study reported that milk and milk products contain high residues of ampicillin (2.44–3.89 µg/L), tetracycline (54.16–220.3 µg/L), oxytetracycline (41.55–160.7 µg/L), and amoxicillin (3.11–5.5 µg/L) [64], as described in Table 4. In camel milk, antibiotic-resistant genes conferring resistance to 18 different antibiotics were detected, with a higher resistance to fluoroquinolones (12.48%) and disinfectants and antiseptics (9%). In total, 80% of microbiome isolates including *K. pneumoniae, E. coli*, and *E. hormaechei*, were resistant to penicillin, tetracyclines, and carbapenems [61].

#### 3.3.3. Environmental Sources

A total of 230 bacterial isolates, including *Enterobacter* sp., *Klebsiella* sp., and *Pantoea agglomerans* were isolated from seawater and demonstrated resistance against penicillin and sulphonamides [51]. A baseline surveillance study investigating the AMR phenotype of *E. coli* in the marine environment reported high resistance rates across different isolation sources and seasons. The resistance rates among seawater isolates ranged from 64% to 89% in summer and 57% to 90% in winter, while the rates in biota isolates were 77% in summer and 88% in winter. In addition, ampicillin resistance was the common phenotype reported from both seawater and biota isolates [53]. Furthermore, *E. coli* samples were isolated from marine sediments and demonstrated a high rate of resistance (95%) against ampicillin, while moderate resistance was observed against cefpodoxime and ciprofloxacin [54]. Moreover, high resistance to cephalosporin (97.1%) was also identified in *E. coli* isolates from samples collected from sewage. Importantly, 91.4% of the isolates were MDR [59]. Moreover, a multicenter study also demonstrated that different bacterial isolates, including *E. coli*, had resistance against ampicillin (31%), azithromycin (15%), cefotaxime (12%), ciprofloxacin (39%). The overall MDR was 33% [24]. The *Acinetobacter* isolates were found to be resistant against colistin (12%) [60] (Table 4).

### 3.4. Transmission Pathways

The prevalence of ARGs such as beta-lactamase (blaCTX-M), quinolone resistance (qnr), multidrug efflux (mdf A), and integron integrase (intI1) has often been documented in clinical, agricultural (animal), and environmental isolates. This clearly shows the risk of cross-domain transmission [52]. The most prevalent ARGs identified in marine sediment in Kuwait were pat A, ErmE, adeF, TaeA, ErmF, tetX, bcrC, mphD, srmB, baeS, Erm30, VIM-7, vanTE, AcrF, tet33, ANT4-1a, adeb, efmA, and rpsl. Moreover, 42% of the identified genes were associated with MDR phenotype [58]. In a similar study, 402 ARGs were identified in marine sediment samples. The most prevalent were those conferring resistance to beta-lactams (37%), macrolides (19%), and tetracycline (7%). The most prevalent genes were tetPA, sul1_1, and blaTEM_1 [56]. In aerosols collected from indoor and outdoor locations in Kuwait, several genes associated with MDR phenotype, fluoroquinolone resistance, and beta-lactam resistance were identified, including the Per-2 group, QnrS, OXA-54, and OXA-55 [57]. Moreover, the resistome profiling in surface sediments revealed a high abundance of resistance elements, including insertion sequences (1782), plasmids (1567), ARGs (609), and integrons (167). Notably, the highest numbers of insertion sequences, plasmids, and integrons were identified in samples collected near an outfall that receives daily emergency hospital waste from a large hospital network [55]. In addition, studies suggested that sewage, marine environment, sediment, poultry, and camel milk may act as sources, reservoirs, and possible direct transmission routes for antimicrobial resistance genes to humans, highlighting the possible pathway for horizontal gene transfer [24,54,58,59,61], as described in Table 5.

### 3.5. Meta-Analysis

#### 3.5.1. Prevalence of Microbes in the Clinical Settings (Human)

**Figure 2:**
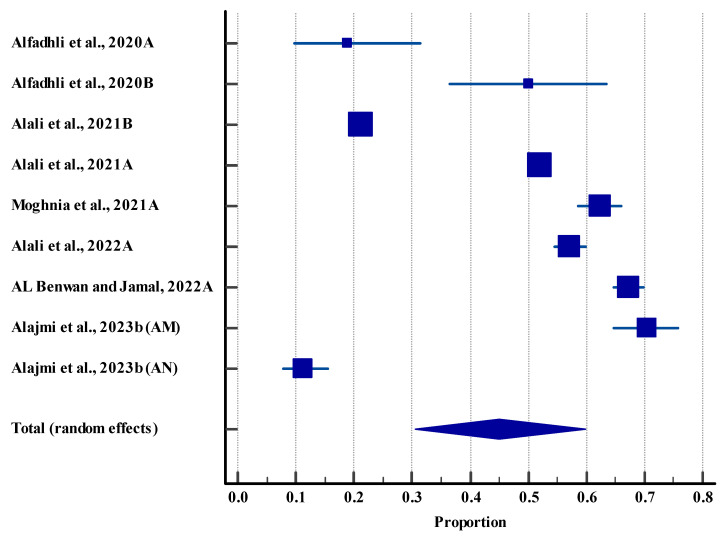
Forest plot for the prevalence of microbes in the clinical settings associated with humans. A indicates *E. coli*, B indicates *K. pneumonia*, AM indicates *E. coli* from mothers, and AN indicates *E. coli* from neonates [40,41,42,43,49,66]. The horizontal line represents the 95% confidence interval for the estimated proportion from individual studies. Blue squares are the point estimates (square size proportional to study weight). The diamond shape represents the pooled estimate from random-effects meta-analysis.

The pooled prevalence effect size was 44.95% with a 95% CI (30.58 to 59.76), indicating a statistically significant difference (*p* < 0.0001). This indicated that nearly half of the study population show the prevalence of *E. coli* and *K. pneumonia*. However, the meta-analysis also revealed a high level of heterogeneity (99.62%) among the included studies (Figure 2). This heterogeneity may be due to the differences in the study design, population, and sample size.

#### 3.5.2. Resistance Rate (Humans)

**Figure 3:**
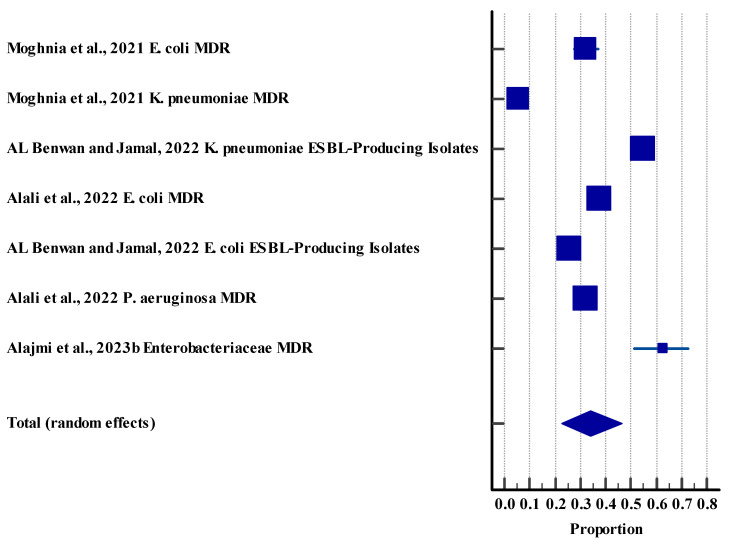
Forest plot for resistance rate among different microbes in humans. MDR: Multiple Drug Resistance, ESBL: Extended-Spectrum β-lactamase [40,42,49,66]. The horizontal line represents the 95% confidence interval for the estimated proportion from individual studies. Blue squares are the point estimates (square size proportional to study weight). The diamond shape represents the pooled estimate from random-effects meta-analysis.

The pooled resistance effect size was 34.05%, 95% CI (22.81 to 46.27), with a significant difference (*p* < 0.0001). Our findings indicated that almost one third of bacterial isolates demonstrated AMR. However, a high heterogeneity (98.94%) was observed among the included studies (Figure 3).

#### 3.5.3. Resistance Rate (Agriculture (Animal))

**Figure 4:**
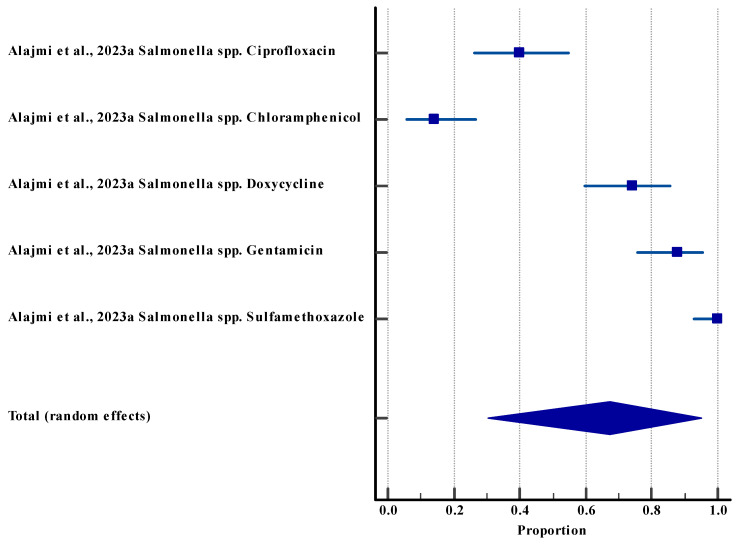
Forest plot for resistance rate among different microbes in animals against different antibiotics [62]. The horizontal line represents the 95% confidence interval for the estimated proportion from in-dividual studies. Blue squares are the point estimates (square size proportional to study weight). The diamond shape represents the pooled estimate from random-effects meta-analysis.

The pooled resistance effect size was 67.42% with a 95% CI (30.30 to 94.93), showing a significant difference (*p* < 0.0001). These outcomes suggest that over half of the samples of Salmonella species showed resistance to different drugs. However, a high level of heterogeneity (97.40%) was observed among the included studies (Figure 4).

#### 3.5.4. Resistance Rate (Environment)

**Figure 5:**
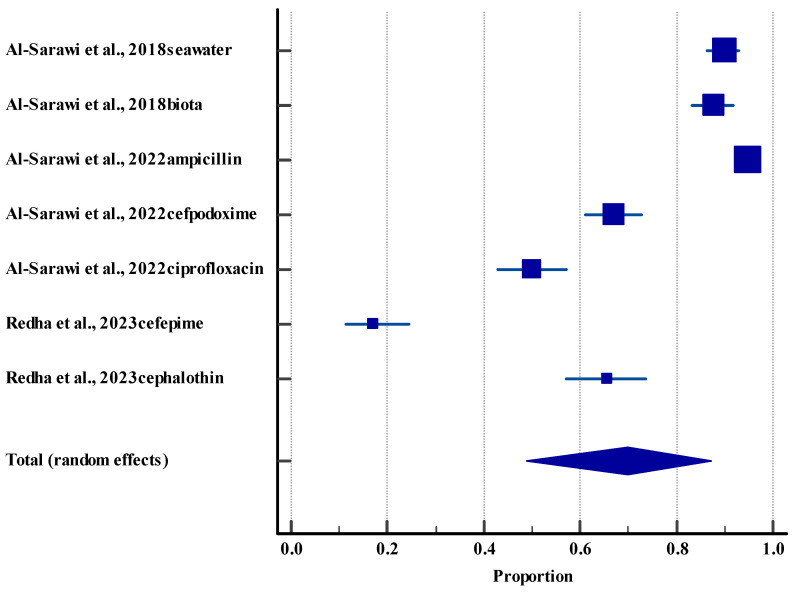
Forest plot showing resistance rate among *E. coli* in the environment against different antibiotics [53,54,59]. The horizontal line represents the 95% confidence interval for the estimated proportion from in-dividual studies. Blue squares are the point estimates (square size proportional to study weight). The diamond shape represents the pooled estimate from random-effects meta-analysis.

The pooled resistance effect size was 69.86%, 95% CI (48.80 to 87.26). These outcomes indicated significant difference (*p* < 0.0001), with almost 70% of the samples of *E. coli* from different sources demonstrating resistance against different types of drugs. However, a high level of heterogeneity (98.78%) was observed among the included studies (Figure 5), which may be due to the different source of samples, types of drugs, and variation in the testing methods.

### 3.6. Methodological Quality Assessment

#### 3.6.1. Clinical (Humans)

**Figure 6:**
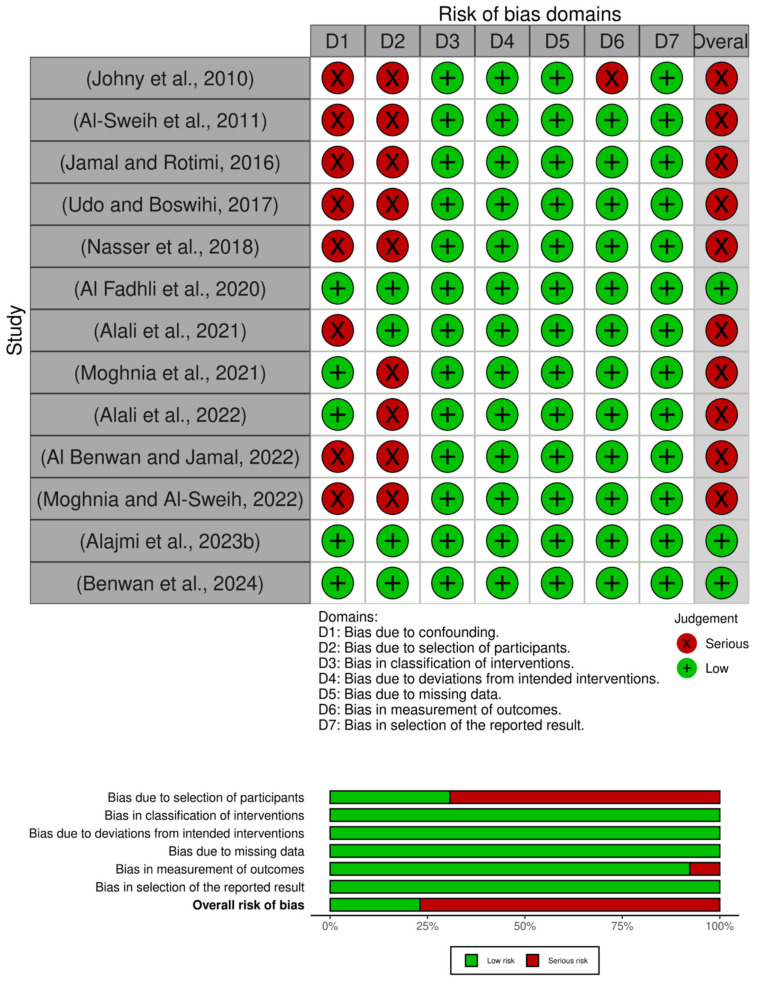
Methodological quality assessment of non-RCTs [40,41,42,43,44,45,46,47,48,49,50,65,66].

Overall, most of the studies demonstrated a serious risk of bias, particularly in the domains of confounding and participant selection, which may influence the interpretability and accuracy of their outcomes. Notably, only three studies, which had an overall low risk of bias in all domains [41,45,49], as described in Figure 6, provide more robust evidence to support the conclusion.

#### 3.6.2. Agriculture (Animal) and Environmental Studies

Overall, agriculture (animal) studies had a low risk of bias in the domains of selection bias, performance bias, detection bias, attrition bias, reporting, and other bias. Two environmental studies [51,53] exhibited a high risk of bias, and one study [58] had some concerns in the domain of selection, as these studies did not provide specific details or criteria regarding the selection of the study samples. One study had a high risk of bias in the domain of performance [53], and the remaining studies had a low risk of bias in all domains, as indicated in Table 6.

### 3.7. Publication Bias

**Figure 7:**
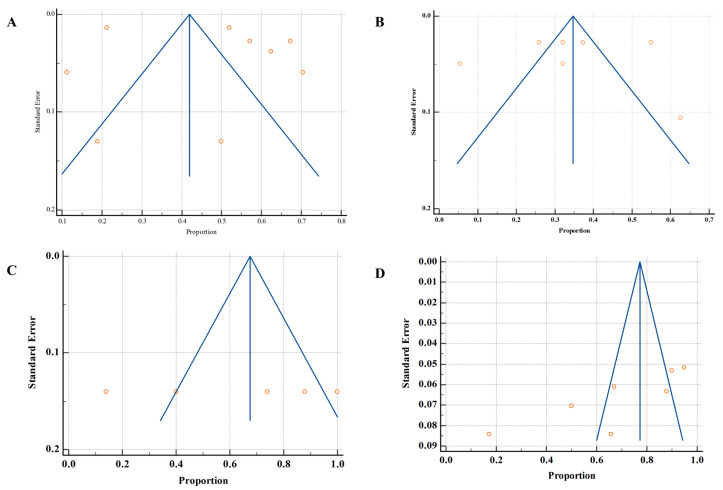
Publication bias. (**A**). Prevalence of microbes in humans, (**B**). AMR in humans, (**C**). AMR in agriculture (animal), (**D**). AMR in the environment. Individual studies shown as orange circles plotted against inverted funnel-shaped confidence limits (blue lines) with the overall pooled estimate indicated by a vertical line.

Funnel plots A and B appear relatively symmetrical, indicating a lower risk of publication bias. In contrast, funnel plots C and D demonstrated asymmetry, with studies skewed to one side, suggesting a potential publication bias (Figure 7).

### 3.8. Certainty of Evidence

Outcomes including the prevalence of microbes in humans and microbial resistance in humans, agriculture (animals), and the environment showed low certainty of evidence due to a high risk of bias, high inconsistency, and publication bias (Table 7).

## 4. Discussion

This is the first systematic review and meta-analysis of integrated studies on AMR through the One Health lens in Kuwait. The aim was to deliver a thorough analysis of AMR status in Kuwait between 2009 and 2024 across the three sectors of humans, animals, and the environment and evaluate the interconnected nature of resistance across these sectors. Therefore, 28 studies were included and analyzed in this systematic review and meta-analysis. The narrative synthesis reported high AMR incidents across human, animal, and environmental domains. Meanwhile, the meta-analysis also indicated a high resistance rate in humans, animals, and the environment.

The increase in antimicrobial selective pressure in the environment is the fundamental driver for the emergence and dissemination of AMR [11]. Selective pressure occurs when the concentrations of antimicrobials favor the survival and proliferation of resistant bacteria over susceptible ones, which creates an evolutionary pressure that enhances the selection and horizontal transfer of ARGs [67]. Importantly, anthropogenic activities across human, animal, and environmental domains accelerate the development of AMR by increasing antimicrobial selective [13].

In our analysis, the pooled resistance rate in the human domain was 34.05%. Globally, the annual rate of antibiotic consumption by humans in 2023 was estimated to be 49.3 billion defined daily doses (DDDs), which increased by 20.9% (40.8 billion DDDs) compared to 2016 [68]. This overprescription of antibiotics presents significant challenges to global healthcare systems. Similarly, Kuwait faces challenges with antibiotic overprescription. Aly et al. [69] reported that 1528 antibiotic prescriptions were granted across nine hospitals in Kuwait over six months, and 47.3% of those did not adhere to the antibiotic policy of the Ministry of Health. Importantly, 25% of prescriptions were administered without clinical indication [69]. Similarly, another study clearly stated that the overuse of antibiotics in Kuwait is associated with a lack of evidence-based practice [70]. The study reported that 50% of patients with upper respiratory tract infections (URTIs) received antibiotic prescriptions treatments; 94% of these prescriptions were non-evidence-based practice according to the National Institute for Health and Care Excellence (NICE) guidelines [70]. The community-level selective pressure in Kuwait also contributes to AMR emergence. A cross-sectional survey of 770 Kuwaiti individuals revealed that 27.5% used antibiotics without prior medical assessment, mainly for viral infections such as the common cold and sore throat. In total, 36% of the individuals with medically prescribed antibiotics did not complete the treatment course, and 47% of participants reported poor understanding of antibiotic action, safety, and resistance mechanisms [71]. Altogether, self-medication, inappropriate use patterns, and a lack of awareness among community contribute to the selective pressure, which drives resistance emergence in community-acquired infections in Kuwait. This pattern of the misuse and overuse of antibiotics across clinical and community settings in Kuwait creates a selective pressure environment, favoring resistant strains and contributing to the 34.05% resistance rate reported in our study among human clinical and community isolates.

The agricultural (animal) domain in this study exhibited the second highest pooled resistance rates (67.42%) among the investigated sectors. *Salmonella* isolates from poultry demonstrated high resistance prevalence; 100% of the isolates were resistant to cefotaxime, 94–100% to ampicillin, and 87.5–96% to amoxicillin. Similarly, different ARGs conferring resistance to 18 different antibiotics were detected in camel milk, and 80% of microbiome isolates were resistant to penicillin, tetracyclines, and carbapenems. These patterns indicate sustained selective pressure from the use of antibiotics in livestock production. In 2019, global antibiotic use in animal production was estimated to be 110,777 tons, and it is expected to increase by 29.5% by 2040 [72]. Almost 90% of antibiotics used in animal production are released unchanged through feces or urine, creating concentrated resistance selection environments in manure, groundwater, and agricultural runoff [73]. The use of antibiotics accelerates AMR development by increasing selective pressure, which supports the growth of resistant bacteria in the gut microbiome, promotes the spread of ARGs through horizontal gene transfer, and induces mutagenesis. Once resistant bacteria and ARGs are excreted in animal waste, resistant bacteria and ARGs are disseminated into the environment [74]. Additionally, antibiotic residues can enter the food chain and environment, further endangering public health. Antibiotic residues in food pose human health risks by promoting exposure to resistant bacteria, such as ciprofloxacin-resistant *Salmonella*, *Campylobacter*, and *E. coli*, reducing the effectiveness of commonly used antibiotics in human medicine. Additionally, residue exposure can disrupt human gut microbiota, increasing vulnerability to infections [75]. Although the data on the use of antimicrobials in animal production in Kuwait are not available, multiple studies reported the contamination of animal food products with AMR, ARGs, and antimicrobial residues. For example, AMR was reported from different *Salmonella* isolates from chicken carcass samples [62]. All *S. enteritidis* isolates were resistant to cefotaxime, while 94% and 87.8% were resistant to ampicillin and amoxicillin, respectively. Additionally, 100%, 94%, and 96% of the *S. typhimurium* isolates were resistant to cefotaxime, ampicillin, and amoxicillin, respectively. Similarly, all *S. Thomson* isolates showed resistance to cefotaxime and 98% of the isolates were resistant to both ampicillin and amoxicillin [62]. Moreover, 80% of microbiome isolates from camel milk were resistant to penicillin, tetracyclines, and carbapenems. Different ARGs conferring resistance to 18 different antibiotics were detected [61]. Sheep meat samples also showed positive results for antimicrobial residues in the muscle (82%), liver (64%), and kidney (100%) [63]. Another study reported that milk and milk products collected from hospital settings contain high residues of ampicillin (2.44–3.89 µg/L), tetracycline (54.16–220.3 µg/L), oxytetracycline (41.55–160.7 µg/L), and amoxicillin (3.11–5.5 µg/L) [64]. The widespread presence of antimicrobial residues, AMR bacteria, and ARGs in edible animal tissues and food products increases selective pressure and accelerates the emergence and spread of resistance beyond the animal domain.

The highest pooled resistance rates in our analysis were detected from the environmental domain, with a rate of 69.86%. The environment serves as a reservoir and a pathway for the transmission of AMR bacteria and ARGs [76]. The overuse of antibiotics in the human and animal domains accelerates the development and release of AMR bacteria and ARGs into the environment. These AMR elements can spread through soil, water, and biofilms via horizontal gene transfer, which then re-enter into the human and animal domains and thereby sustain the intersectoral cycle of AMR emergence and transmission [76]. The release of antimicrobial residues, heavy metals, and disinfectants into the environment further increases selective pressure on the microbial community microorganisms [11]. The marine environment plays a dual role in the context of AMR. First, it serves as a reservoir for AMR and ARGs [77]. Second, it acts as a sink for pharmaceuticals and heavy metal residuals that exert selective pressure on bacterial communities [52]. Collectively, such dual roles promote the development and dissemination of resistance traits.

Multiple studies confirmed the contamination of the marine environment in Kuwait with resistant elements (AMR bacteria and ARGs) [24,51,52,53,54,55,58,60], antimicrobial residuals [78] and heavy metals [79]. For example, the genomic profile of AMR *E. coli* isolates from marine samples detected 33 ARGs encoded resistance to 7 antibiotic classes [52]. Al-Otaibi et al. [51] reported the high prevalence of resistance among bacterial isolates, including *Enterobacter* sp., *Klebsiella* sp., and *Pantoea agglomerans*, isolated from seawater samples from a Kuwait bay against penicillin and sulphonamides. In another study, high resistance rates among *E. coli* isolates were reported across different isolation sources (seawater and bivalves) and seasons (summer and winter) [53]. The resistance rates ranged from 64% to 89% and 57% to 90% during summer and winter, respectively. Meanwhile, the resistance rates of bivalve isolates were 77% during summer and 88% in winter. Ampicillin resistance was the common phenotype reported from both seawater and bivalve isolates [53]. Furthermore, *E. coli* isolates from marine sediments demonstrated a high resistance prevalence (95%) against ampicillin, while moderate resistance was observed against cefpodoxime and ciprofloxacin [54]. Moreover, a multicenter study reported high resistance prevalence among bacterial isolates against ampicillin (31%), azithromycin (15%), cefotaxime (12%), ciprofloxacin (39%). Overall MDR prevalence was high (33%) [24]. In a different study, 12% of the *Acinetobacter* isolates from seawater samples were resistant to colistin [60]. Habibi et al. [58] identified hundreds of ARGs from surface sediments collected from 12 sites adjacent to and remote from stormwater outfalls. The metagenomic analysis captured 402 ARGs belonging to 13 gene families. These ARGs encode resistance to 34 antibiotics of different classes. Interestingly, the number of resistant elements detected at a site adjacent to an emergency hospital waste outfall was the highest compared to other sampling sites [58]. This site was previously reported by others to contain the highest concentrations of pharmaceutical and antibiotic residues [79]. In a subsequent study, the same group used shotgun metagenomic sequencing to profile the resistome of the microbial community in the coastal sediments [55]. The aligned sequence annotated to ARGs (609) plasmids (1567), integrons (167) and insertion sequence (1782). The distribution of those resistant elements varied across the sampling sites. Importantly, the highest numbers were recorded at a site near an outfall that receives emergency hospital waste [55]. Gevao et al. [78] detected 13 antibiotics from different classes in seawater samples collected from Kuwait’s coastal waters. The mean concentrations of those varied from 39 ng/L (erythromycin) to 657 ng/L (ciprofloxacin). Importantly, the comparative assessment showed that concentrations of some of these antibiotics were significantly higher than those reported in different water bodies in some other countries [78]. Additionally, heavy metals were detected in marine sediments in Kuwait, with some exceeding national sediment quality guidelines [79].

In addition to the marine environment, the sewage system in Kuwait is also contaminated with antimicrobial residuals, heavy metals, and AMR bacteria. More than 90% of the municipal wastewater in Kuwait is treated by seven wastewater treatment plants (WWTPs). Approximately 90% of the wastewater is treated, and the remainder is discharged untreated into the sea [80]. Antibiotics and heavy metals have been detected in the streams of different WWTPs in Kuwait [81,82]. Such contaminations create persistent selective pressure on the microbiota of the sewage system, which promotes the emergence and dissemination of AMR. A total of 13 antibiotics belonging to six classes were reported in the streams of Umm Al Hayman and Kabd WWTPs [81]. The highest average concentration reported in the influent of both WWTPs combined was clarithromycin (770.5 ng/L). On the other hand, the highest average reported from effluent streams combined was sulfamethoxazole (275.0 ng/L) [81]. Unfortunately, these contaminants spread beyond these plants and were identified in the marine water [78]. In addition, heavy metals were also detected in WWTP. Al Enezi et al. [82] reported that raw wastewater and sludge produced at the Ardiya WWTP contain different concentrations of seven heavy metals. Out of those, the levels of copper, cadmium and mercury were above the accepted concentrations for agricultural use. Additionally, five of these metals were detected in the tissue of some crops, some at higher than the typical concentrations [82]. AMR bacteria have also been detected in wastewater in Kuwait. Redha et al. [59] characterized the AMR profile of *E. coli* isolated from sewage samples. Most of the isolates (91.4%) showed resistance to 3 to 12 classes and were thus classified as MDR. Importantly, two isolates (1.4%) exhibited an extreme drug-resistant (XDR) phenotype, resistant to 13–14 classes [59]. Collectively, all these studies confirmed that the marine environment and the sewage system in Kuwait are heavily contaminated with AMR bacteria, ARGs, antimicrobial residues, and heavy metals, which all impose selective pressure on marine microbiota and facilitate the development and dissemination of AMR.

In this study, we also found that there are important connections in resistant elements between humans, agriculture (animals), and the environment, showing that these sectors share resistant elements. Common resistant elements, including beta-lactamase (blaCTX-M), quinolone resistance (qnr), and integron integrase (intI1) were identified across all domains. This genetic overlap demonstrates that selective pressure in one domain rapidly affects others through horizontal gene transfer and cross-contamination.

To overcome the complex problem of AMR in Kuwait, which is triggered by the interconnectedness of all three sectors, it is important to approve a One Health strategy among different government bodies. As the One Health approach can assist and encourage future linkages between human, animal, and environmental frameworks, particularly in addressing zoonotic diseases, the environment also needs to be focused on due to a high rate of resistant microbes. Unfortunately, none of the included studies focused on the multiple sectors or used the One Health approach in Kuwait. To successfully lower resistance, an action plan should be created and implemented. Understanding how different health sectors work together shows the importance of using collaborative and multidisciplinary approaches to deal with the growing problem of resistance.

The strength of the present review is that it offers an exhaustive examination of the resistance pattern of microbes in the context of One Health. However, its limitations should also be addressed. For instance, several studies used cross-sectional or descriptive designs, making it difficult to distinguish between causal relationships and mere statistical associations. Most of the studies did not present detailed resistance profiles or genetic data, and sample sizes and settings were different. There was not much direct evidence of transfer between domains, even though there was repeated genetic overlap. No information regarding the One Health approach was included. Notably, a high level of heterogeneity was observed among the studies, which may be due to the included isolates, study population, study design, and multiple data points from the same study. In addition, low certainty of evidence was observed. Most non-RCTs had a high risk of bias, and the included studies also exhibited publication bias. Additionally, the outcomes demonstrated low certainty of evidence. Therefore, the outcomes of the present review should be used with caution. As no study used the One Health approach, the studies only provided insights into the prevalence of AMR and the genomics of resistant genes within a particular domain. Future studies should focus on the One Health approach.

## 5. Conclusions

This review focused on the AMR situation in the state of Kuwait from a One Health perspective. In the human domain, Gram-negative isolates, such as *E. coli*, *K. pneumoniae*, *P. aeruginosa*, and *Acinetobacter baumannii*, were the most prevalent resistant microbes. In the agriculture (animals) domain, *Salmonella* isolates had a high resistance to cefotaxime, ampicillin, and amoxicillin. Samples from the environment demonstrated the presence of widespread ARGs. Meta-analytic analysis also demonstrated a high rate of resistance in humans in clinical settings as well as in the agricultural (animal) and environmental sectors. The development of resistance to different drugs, including carbapenems, highlights the importance of the current situation of AMR and how crucial it is for governments to put an effective action plan into place and interlink these sectors to overcome the problem of AMR. Altogether, these detailed results showed that a coordinated One Health approach is critical to addressing the problem of AMR in Kuwait.

## Figures and Tables

**Table 1:** Eligibility criteria for the selection of studies.

Criteria Category	Inclusion Criteria	Exclusion Criteria
Geographic Focus	Studies included data specifically from Kuwait	Studies did not include data from Kuwait
Study Topic	Studies examined antibiotic resistance patterns, prevalence, or transmission	Studies that do not examine antibiotic resistance patterns, prevalence, or transmission
Study Design	Primary research studies (randomized, observational, experimental, surveillance)	Studies that are not primary research
Study Setting	Studies examined samples from at least one of the following: human populations, agricultural practices/livestock, or the environment	Studies that do not examine samples from human populations, agricultural practices/livestock, or environmental sources
Data Type	Studies included antimicrobial susceptibility or resistance data	Studies that do not include antimicrobial susceptibility or resistance data
Study Scope	Studies examined more than a single case report	Studies that examine only a single case report
Timeline	Studies published between 2009 and 2024	Studies published outside the 2009–2024 timeframe
Language	In English	Not in English

**Table 2:** General characteristics of included studies.

Study	Study Setting	Primary Focus	Study Design	Sample Size
[51]	Environmental (marine)	Pathogenic bacteria in seawater	Environmental sampling	230 bacteria
[46]	Clinical (hospital)	*Streptococcus pneumoniae*	Retrospective	1353 strains
[44]	Clinical (hospital)	Tigecycline/colistin resistance in *Acinetobacter*	Cross-sectional Surveillance	250 isolates
[65]	Clinical (hospital/community)	*Clostridioides difficile* resistance	Surveillance	146 isolates
[48]	Clinical (hospital)	MRSA resistance trends	Surveillance	6922 isolates
[53]	Environmental (marine)	Resistance in *Escherichia coli* from seawater/biota	Environmental sampling, surveillance	598 isolates
[50]	Clinical (hospital)	Genomics of MDR *Acinetobacter baumannii*	Not clearly stated	6 isolates
[41]	Clinical (hospital)	Carbapenem-resistant *Enterobacteriaceae*	Retrospective	590 sample
[43]	Clinical (hospital)	Antimicrobial resistance prevalence in Gram-negative bacteria	Retrospective descriptive, surveillance	5290 isolates
[66]	Clinical (hospital/community)	Carbapenem-resistant *Enterobacteriaceae*	Prospective	681 isolates
[42]	Clinical (hospital)	Resistance in *E. coli* and *Pseudomonas aeruginosa* pre/during COVID-19	Observational (retrospective)	1303 isolates
[40]	Clinical (hospital)	Resistance in gram-negative bacteria	Retrospective	9742 urine samples
[54]	Environmental (marine sediment)	Resistance in *E. coli* from sediment	Environmental sampling	395 isolates
[47]	Clinical (hospital)	MDR *E. coli* and *Klebsiella pneumoniae*	Retrospective	2 samples
[58]	Environmental (marine sediment)	ARGs in marine sediments	Environmental sampling, cross-sectional	12 samples
[24]	Environmental (marine)	AMR monitoring in GCC marine environment	Surveillance, environmental sampling	560 isolates
[63]	Agricultural (sheep meat)	AMR residues in sheep carcasses	Observational (retrospective), surveillance	450 samples
[59]	Environmental (sewage)	MDR/XDR *E. coli* in sewage	Environmental sampling, observational (retrospective)	140 isolates
[52]	Environmental (marine)	Genomic AMR in *E. coli*	Surveillance, environmental sampling	395 isolates
[62]	Agricultural (poultry)	*Salmonella* in broiler chickens	Observational (retrospective)	4128 samples
[49]	Clinical (hospital, neonates)	MDR *Enterobacteriaceae* in neonates/mothers	Cross-sectional	484 samples
[55]	Environmental (coastal sediment)	Microbiome, metabolic function, resistome	Environmental sampling	12 samples
[56]	Environmental (sediment)	HT-qPCR vs. metagenomics for resistome	Surveillance, environmental sampling	Not stated
[57]	Environmental (aerosols)	ARGs in aerosols	Environmental sampling	Not stated
[61]	Agricultural (camel milk)	Resistome in camel milk	Environmental sampling	8 samples
[64]	Agricultural (milk and milk-based products)	Antibiotic resistance	Not clearly stated	200 samples
[60]	Environmental (marine sediment)	Antibiotic resistant genes and fecal sterols	Not clearly stated	20 samples
[45]	Clinical (hospital)	Anaerobes	Prospective	2317 isolates

Abbreviations: AMR: Antimicrobial Resistance, ARGs: Antimicrobial Resistance Genes, HT-qPCR: High-Throughput Quantitative Polymerase Chain Reaction, MDR: Multidrug Resistance, XDR: Extensively Drug Resistance, GCC: Gulf Cooperation Council, COVID–19: Coronavirus disease–19.

**Table 3:** Summary of study characteristics.

Category	Subcategory/Detail	Number of Studies/Details	Notes
Study Setting	Clinical	11	Some studies overlap categories
	Environmental	11	
	Agricultural (animal)	4	
	Mixed settings (Clinical/Community)	2	
	Regional (multi-country)	1	
	Single country	27	
Primary Focus	Antimicrobial resistance prevalence/trends (including specific organisms)	23	e.g., *E. coli*, MRSA, *Salmonella* spp.
	Resistome or antimicrobial resistance genes	5	
Study Design	Surveillance design (including environmental surveillance and sampling)	11	Some overlap with environmental sampling
	Retrospective/observational	9	
	Cross-sectional	3	
	Prospective	2	
	Not clearly stated	3	No clear design in study/abstract; however, we consider these studies non-randomized
Sample Size	Sample size reported	26	Ranged from 2 to 6922 (isolates/samples/individuals)
	Sample size not reported	2	Not clearly mentioned in study/abstract
One Health approach	None	0	None of the studies used the One Health approach or integrated surveillance program for AMR in humans, agriculture (animals), and environment

Abbreviations: MRSA: Methicillin-Resistant *Staphylococcus aureus*, AMR: Antimicrobial Resistance, spp.: Species.

**Table 4:** Summary of resistant patterns among bacterial species in different domains of detection.

Bacterial Species	Resistance Pattern	Prevalence	Domain of Detection	References
*E. coli*	Multidrug resistance, extended-spectrum beta-lactamase, carbapenem, and ampicillin resistance	Multidrug resistance: 38.7–91.4%; extended-spectrum beta-lactamase: up to 62.4%; carbapenem: up to 10%	Clinical, sewage, marine (seawater and biota), sediment	[42,49,59,66]
*K. pneumoniae*	Multidrug resistance, extended-spectrum beta-lactamase, carbapenem resistance	Multidrug resistance: up to 36.2%; extended-spectrum beta-lactamase: up to 91%; carbapenem: up to 38%	Clinical, environmental	[40,42,49,53]
*P. aeruginosa*	Multidrug resistance, carbapenem resistance	Multidrug resistance: up to 32.1%; carbapenem: variable	Clinical	[42,49]
*Acinetobacter baumannii*	Multidrug resistance, carbapenem, and colistin resistance	Multidrug resistance: up to 88.4%; carbapenem: up to 25.2%; colistin: up to 12%.	Clinical	[43,44]
Methicillin-resistant *S. aureus*	Multi-antibiotic resistance	32–60.7% methicillin-resistant *Staphylococcus aureus*; resistance to multiple agents	Clinical	[48]
*Salmonella*	Multi-antibiotic resistance	High resistance to multiple antibiotics	Agriculture (animal [poultry])	[62]
Antimicrobial resistance genes (environmental)	Beta-lactams, fluoroquinolones, aminoglycosides, etc	402 antimicrobial resistance genes in sediment; 98% of *E. coli* in sediment are resistant	Marine, sediment, aerosols	[24,56,57,58]

**Table 5:** Common resistance mechanisms.

Resistance Type	Human Health Impact	Agricultural Link	Environmental Presence
Extended-spectrum beta-lactamase (e.g., CTX-M-15)	Limits treatment options for infections; associated with outbreaks	Detected in poultry, camel milk	Found in sewage, marine, and sediment
Carbapenemases (e.g., OXA-48, NDM-1)	Associated with multidrug-resistant/extensively drug-resistant infections, high mortality	Detected in the food animal regionally	Detected in environmental samples
Colistin resistance (e.g., mcr genes) Fluoroquinolone resistance (e.g., qnr genes)	Last-resort antibiotic compromised Reduces efficacy of key antibiotics	Detected in the food chain regionally Detected in poultry, camel milk	Detected in environmental samples found in marine and sediment
Integrons (e.g., intI1)	Facilitates horizontal gene transfer	Detected in food/environment	Found in marine, sediment

**Table 6:** Methodological quality assessment of agriculture (animal) and environmental studies.

Study ID	Selection Bias	Performance Bias	Detection Bias	Attrition Bias	Reporting Bias	Other Bias
Animal studies						
[63]	Low	Low	Low	Low	Low	Low
[62]	Low	Low	Low	Low	Low	Low
[61]	Low	Low	Low	Low	Low	Low
[64]	Low	Low	Low	Low	Low	Low
Environmental studies						
[51]	High	Low	Low	Low	Low	Low
[53]	High	High	Low	Low	Low	Low
[54]	Low	Low	Low	Low	Low	Low
[58]	Some concerns	Low	Low	Low	Low	Low
[24]	Low	Low	Low	Low	Low	Low
[59]	Low	Low	Low	Low	Low	Low
[52]	Low	Low	Low	Low	Low	Low
[57]	Low	Low	Low	Low	Low	Low
[56]	Low	Low	Low	Low	Low	Low
[55]	Low	Low	Low	Low	Low	Low
[60]	Low	Low	Low	Low	Low	Low

**Table 7:** Certainty of evidence using the GRADE assessment framework.

Outcomes	Studies	Risk of Bias	Inconsistency	Indirectness	Imprecision	Publication Bias	Effect Size	Certainty of Evidence
Prevalence of microbes in humans	9	High	Serious (I^2^ = 99.62%)	Not serious	Not serious	No	44.95%, 95% CI (30.58 to 59.76),	Low θθ
Resistance rate (humans)	7	High	Serious (I^2^ = 98.94%)	Not serious	Not serious	No	34.05%, 95% CI (22.81 to 46.27)	Low θθ
Resistance rate (animal)	5	Low	Not serious (I^2^ = 97.40%)	Not serious	Not serious	Yes	67.42%, 95% CI (30.30 to 94.93)	Low θθ
Resistance rate (environment)	4	Low	Serious (I^2^ = 98.78%)	Not serious	Not serious	Yes	69.86%, 95% CI (48.80 to 87.26)	Low θθ

## Data Availability

No new data were created or analyzed in this study. Data sharing is not applicable to this article.

## Supplementary Materials

The following supporting information can be downloaded at: https://www.mdpi.com/article/10.3390/life15091344/s1, Table S1: PRISMA 2020 Checklist.

## Abbreviations

The following abbreviations are used in this manuscript:

AMR	Antimicrobial Resistance
ARGs	Antibiotic Resistance Genes
blaCTX-M	Beta-Lactamase
BMC	BioMed Central
CA	Community-Acquired
CDIs	*C. difficile* Infections
COVID-19	Coronavirus disease
CRE	Carbapenem-Resistant *Enterobacteriaceae*
DDDs	Defined Daily Doses
ESBL	Extended-Spectrum β-Lactamase
FAO	Food and Agriculture Organization
GCC	Gulf Cooperation Council
GRADE	Grading, Reporting, Assessment, Development, and Evaluation
HA	Healthcare-Associated
intI1	Integron Integrase
mdf A	Multidrug Efflux
MDR	Multiple Drug Resistance
MENA	Middle East and North Africa
MRSA	Meticillin-Resistant *Staphylococcus aureus*
NAPs	National Action Plans
Non-RCTs	Non-Randomized Controlled Trials
OIE	World Organization for Animal Health
PRISMA	Preferred Reporting Items for Systematic Reviews and Meta-Analyses
Qnr	Quinolone Resistance
ROBINS-I	Risk of Bias In Non-Randomized Studies—Interventions
WHO	World Health Organization
WWTPs	Wastewater Treatment Plants
XDR	Extensively Drug Resistance
YLDs	Years Lived with Disability
